# Respiratory Syncytial Virus Suppression through Public Health and Social Measures, Hong Kong, China, 2020–2023

**DOI:** 10.3201/eid3208.251887

**Published:** 2026-08

**Authors:** Yanfang Xu, Weijia Xiong, Xiaotong Huang, Benjamin J. Cowling, Tim K. Tsang

**Affiliations:** WHO Collaborating Centre for Infectious Disease Epidemiology and Control, The University of Hong Kong School of Public Health, Hong Kong, China

**Keywords:** respiratory syncytial virus, RSV, viruses, respiratory infections, public health measures, social measures, Hong Kong

## Abstract

Public health measures during COVID-19 were associated with reduced respiratory syncytial virus cases in Hong Kong, China. Mask wearing was associated with a 35% reduction; avoidance behaviors and hand hygiene were also associated with reductions. After the mask mandate was lifted in March 2023, we observed resurgence in RSV activity.

Respiratory syncytial virus (RSV) is a major cause of acute lower respiratory tract infections in infants, young children, and older adults. In Hong Kong, China, RSV circulates year-round with typical seasonal patterns (*1*). The COVID-19 pandemic altered RSV epidemiology through stringent public health and social measures (PHSMs), including masking, hand hygiene promotion, and gathering restrictions. Those interventions nearly eliminated RSV circulation until mask mandates were relaxed in March 2023; RSV incidence resurged by mid-2023 (*2*). Environmental factors, particularly temperature and humidity, might also influence RSV transmission. We analyzed RSV transmission in Hong Kong during 2005–2024 using epidemiologic, behavioral, and meteorologic data and examined the combined effects of PHSMs.

## The Study

We estimated weekly RSV activity by multiplying influenza-like illness (ILI) consultation rates by the proportion of respiratory specimens testing positive for RSV, adjusted for population size (data from Centre for Health Protection, Hong Kong). We used the case estimates as a proxy measure of the weekly incidence of infections in the community (*3*). We obtained meteorologic variables, including temperature, wind speed, humidity, pressure, and rainfall, from the Hong Kong Observatory.

We derived PHSM data from cross-sectional telephone surveys of Hong Kong adults conducted during 2020–2023, measuring adoption of 8 behaviors across 2 categories: social distancing (avoiding crowded areas, going out, healthcare facilities, and social gatherings) and personal protection (mask use, handwashing, and protective practices with shared objects) (Appendix). The survey, which was approved by the Institutional Review Board of the University of Hong Kong, captured individual preventive behaviors but not policy-level interventions such as school closures or traveler quarantine. Among the survey-captured behaviors, mask use was the only behavior directly linked to a territorywide mandate; other PHSMs were gradually relaxed beginning in mid-2022, and the mask mandate was lifted on March 1, 2023 (*4*).

We modeled RSV activity using Bayesian negative binomial regression (*5*). The outcome was the log-transformed RSV activity at week t. Baseline covariates included RSV activity at week t − 1 to account for autocorrelation, and offset using population size at week t to account for change of population size over years (Appendix). We categorized survey variables into social distancing behaviors and personal protective behaviors. Model selection proceeded in 2 stages: we identified optimal combinations of PHSM variables (1–3 variables with lags of 0–2 weeks), then added meteorologic variables (log-transformed and quadratic-transformed temperature and humidity) (Appendix Figure 2). We compared models using leave-one-out cross-validation; we selected those within 2 SEs of the highest expected log predictive density (*6*), expressing coefficients as percent change in risk using the equation 100% × (exp(β) – 1) to represent the proportional change in RSV activity when a behavior changed from no to full adoption. Those estimates reflect within-period proportional changes in RSV activity associated with variation in survey-measured behaviors, rather than comparisons with the 2023 severe season or a prepandemic baseline.

**Figure 1:**
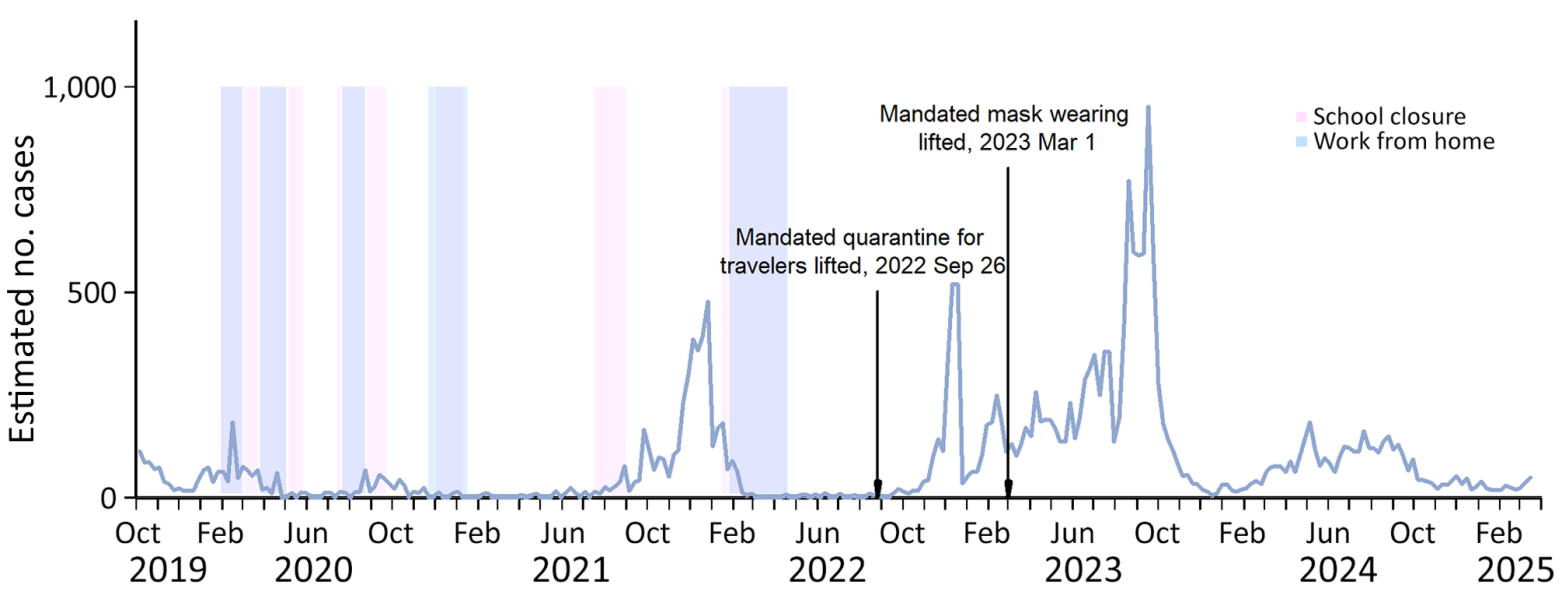
Weekly respiratory syncytial virus activity and several preventive measures against COVID-19 during 2019–2025 in study of respiratory syncytial virus suppression through public health and social measures, Hong Kong. Activity is indicated by estimated number of cases.

Annual RSV epidemics occurred from late autumn to early spring until 2019 (Appendix Figure 1), but activity declined sharply during the COVID-19 pandemic, reaching a minimum during December 2020–July 2021. In November–December 2021, there was an RSV epidemic during the interval between 2 periods of school closures (Figure 1). After the mandated quarantine for travelers was lifted in September 2022 (*7*), RSV resurged and reached a peak in late 2022. After the mask mandate was lifted in March 2023, RSV activity peaked at ≈2 times the magnitude of the previous 2 epidemics. Self-reported mask use remained >90% through early 2023 but declined markedly after the mask mandate was lifted in March 2023; we observed similar downward trends for other PHSMs (Appendix Figure 4). Negative binomial regression confirmed substantial (62%–76%) reductions in RSV activity during the pandemic period (Appendix Table 3).

**Figure 2:**
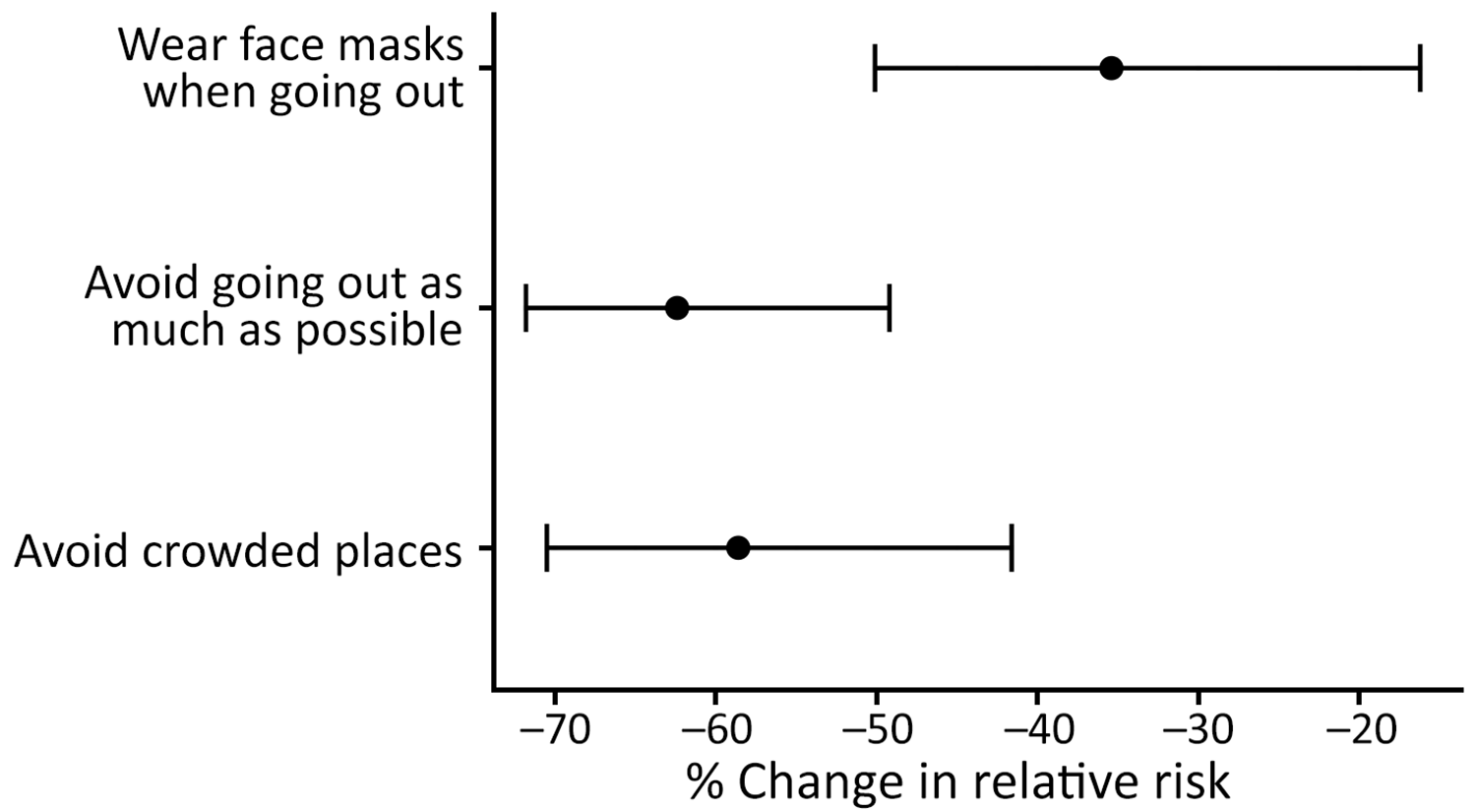
Percentage change in relative risk for several preventive behaviors in study of respiratory syncytial virus suppression through public health and social measures, Hong Kong, China, 2020–2023. Dots represent percentage change; error bars indicate 95% CIs.

We conducted regression testing for association between PHSMs and RSV activity (Figure 2). Mask wearing was associated with a 35% (95% CI 16%–50%) reduction in RSV activity. Avoiding going out (62% [95% CI 49%–72%] reduction), avoiding crowded places (59% [95% CI 42%–71%] reduction), and washing hands immediately after going outside (40% [95% CI 13%–58%] reduction) were also associated with reduced RSV activity. Alternative model specifications were consistent with a protective association of mask wearing (32%–43% reduction across models) and the importance of combined preventive measures (Appendix Figure 3). All PHSM variables selected in the models were 2-lag transformed.

After adjustment for meteorologic variables, the associations between behavioral interventions and RSV activity remained statistically significant and of similar magnitude. In the best weather-adjusted model, RSV activity increased by 6% (95% CI 3%–9%) for every 10% increase in relative humidity. Temperatures were less consistent across model specifications (Appendix Tables 1, 2).

Our analysis demonstrated that mask wearing was associated with a 35% reduction in RSV transmission, consistent with observations for other respiratory pathogens. That finding builds on previous work showing a 25% reduction for influenza (*4*) and aligns with laboratory studies demonstrating that masks reduce droplet emission by up to 90% (*8*). Similar findings across respiratory viruses support mask recommendations during RSV epidemics.

Other protective behaviors, such as avoiding crowded places and limiting outings, were also associated with reduced RSV activity. The combined implementation of those measures during Hong Kong’s COVID-19 restrictions coincided with dramatic RSV suppression, including >80% declines in pediatric hospitalizations compared with prepandemic years (*9*). The interruption of RSV circulation might have increased population susceptibility (accumulated immune debt), which might have made the association between masking and RSV activity seem larger (*10*).

The major RSV epidemic in mid-2023, shortly after the mask mandate was lifted, illustrates the rapid return of respiratory virus transmission after relaxation of protective measures (*11*). Meteorologic factors, particularly relative humidity, were also associated with transmission patterns in subtropical Hong Kong. Higher humidity was associated with increased RSV activity; possible causes were enhanced viral stability and behavioral changes that increase indoor crowding (*12*).

## Conclusions

Our findings have important policy implications for RSV control. Although populationwide mask policies may not be sustainable long term, targeted masking during peak epidemic periods, particularly in healthcare settings, schools, kindergartens and daycare centers, and residential care facilities, was a practical intervention. Combined with enhanced surveillance, our findings suggest that timely populationwide mask mandates could help prevent severe off-season epidemics while minimizing disruption and healthcare system strain (*13*). Compared with reports from Europe and North America (*14*), Hong Kong showed a distinct postpandemic RSV pattern, which might partly reflect differences in mask-wearing adherence, school closure policies, and accumulated immune debt.

Our study did not account for potential changes in RSV ascertainment during the pandemic because of reduced testing and altered healthcare-seeking behavior. We did not conduct age-stratified analysis despite the greater burden of severe RSV disease in infants, young children, and older adults (*15*). As an ecologic study using aggregate surveillance and survey data, our findings reflect population-level associations and cannot establish causal effects of specific interventions at the individual level. Our analysis reflects RSV transmission during the COVID-19 period we studied. Subsequent introduction of RSV vaccines and immunoprophylaxis could change the relative role of PHSMs in current RSV prevention.

Appendix Additional information from study of respiratory syncytial virus suppression through public health and social measures, Hong Kong, 2020–2023.
