# Supplementary material for: Respiratory Syncytial Virus Suppression through Public Health and Social Measures, Hong Kong, China, 2020–2023
Source: Emerg Infect Dis. 2026 Aug;32(8):1315–8. doi: 10.3201/eid3208.251887 (PMC13426844; doi:10.3201/eid3208.251887)
Supplement: Appendix — Additional information from study of respiratory syncytial virus suppression through public health and social measures, Hong Kong, 2020–2023. [file 25-1887-Techapp-s1.pdf]

Article DOI: https://doi.org/10.3201/eid3208.251887

EID cannot ensure accessibility for supplementary materials supplied by authors. Readers who have difficulty accessing supplementary content should contact the authors for assistance.

Respiratory Syncytial Virus Suppression through Public Health and Social Measures, Hong Kong, China, 2020–2023

Appendix

Details of Cross-Sectional Telephone Surveys

PHSM data were derived from cross-sectional telephone surveys of Hong Kong adults (2020–2023), measuring adoption of eight protective behaviors: avoiding crowded areas, avoiding going out, avoiding healthcare facilities, avoiding social gatherings, wearing face masks when going out, washing hands immediately after going outside, washing or sanitizing hands immediately after touching shared objects, and avoiding touching or using protective measures with shared objects.

The PHSM behavioral data in our study were obtained using a similar serial population-based telephone survey platform as Cowling et al. (*1*). This methodology has been widely validated in Hong Kong and was also used during SARS and the 2009 H1N1 pandemic (*2*–*4*). Participants were recruited through repeated cross-sectional telephone surveys using random-digit dialing of both landline and mobile telephone numbers generated by a computer-based randomization system. Trained interviewers placed calls during both working and non-working hours to reduce overrepresentation of non-working populations. Eligible respondents were adults aged ≥18 years who could communicate in Cantonese or English. Surveys were conducted in repeated rounds over time, with a new sample drawn for each round. The look-back period for behavioral questions was 1 week. For household contacts, the household member with the nearest upcoming birthday was invited to participate rather than the person who initially answered the phone.

The questionnaire collected information on risk perceptions, attitudes toward COVID-19, and preventive behaviors, including hygiene practices, mask use, and reductions in social contact. Across all survey rounds conducted from 2021 to 2023, 5% of dialed numbers were invalid or non-residential, 67% were unreachable (including busy lines, call blocking, answering devices, and language barriers), and 25% were refusals, break-offs, or unavailable respondents. Overall, 3% of dialed numbers resulted in completed interviews, yielding 64,462 completed interviews in total. Ethical approval was obtained from the Institutional Review Board of the University of Hong Kong, and verbal informed consent was obtained from all participants.

PHSMs were gradually relaxed from mid-2022, with the mask mandate lifted on March 1, 2023. Pre-2020 baselines were imputed using the latest survey (December 3, 2023), assuming willingness to adopt self-protective behaviors had returned to pre-pandemic levels.

Bayesian Generalized Linear Model for RSV Activity

We fitted a Bayesian generalized linear model with a negative binomial distribution for RSV activity (*5–7*).

$$C_{t+1} \sim NB\left(\lambda_t, \theta\right)$$

Multiplicative model:

$$\log \lambda_t = \beta_0 + \beta \log\left(C_t + 1\right) + \alpha \log\left(X_t + 1\right) + \log N_t$$

In the multiplicative model, the C_t presents the RSV case in time t, X_t is the covariate matrix in time t, including weather, pollution, and PHSMs variables, and N_t present the Hong Kong population in time t. We set the same population for each year.

For estimation, we used a Bayesian statistical framework. The Bayesian framework employs a comprehensive probabilistic model that captures both the uncertainty in an outcome variable y given unknown parameters θ and the prior uncertainty about the parameters θ themselves. For the prior beliefs about the parameters and the posterior beliefs about the parameters, they have the relationship as below:

$$p(\theta \mid y) = \frac{p(y \mid \theta) p(\theta)}{p(y)}$$

For regression models, the Bayes' theorem could be explained by the equation:

$$p(\theta \mid y, x) \propto p(y \mid \theta, x)\, p(\theta \mid x)$$

Here, parameters θ were given weakly informative prior distribution, in which regression parameters have multi-variate normal distribution with mean 0 and variance 1 as the prior distribution, and the dispersion parameters have an exponential distribution with a mean of 1 as the prior distribution.

Besides, we conduct the estimate based on a weak informative prior distribution for the N(0,0.2). Model certification is defined by estimating each model's expected log predictive density (elpd_loo) via Pareto-smoothed importance sampling leave-one-out cross-validation (PSIS-LOO), followed by pairwise comparisons (using the R package loo) (*8–10*).

In our regression models, the estimated coefficients β for the survey variable were transformed using the expression exp(β)-1. This transformation facilitates interpretation by converting the log–linear effect into a relative change on the original scale of the outcome. Specifically, while exp(β) represents the multiplicative effect on the expected count (i.e., the risk ratio), subtracting one yields the proportional change relative to the baseline. For example, a value of −0.35 for the transformed mask coefficient indicates a 35% decrease in the outcome associated with a one-unit increase in the mask wearing. For clarity, we refer to these transformed coefficients as the "relative risk change (RRC)." This terminology emphasizes that the measure reflects the proportional change in risk (or incidence) rather than the raw regression coefficient. The RRC provides an intuitive interpretation of model results, particularly when comparing the magnitude and direction of effects across covariates.

References

1. Cowling BJ, Ali ST, Ng TWY, Tsang TK, Li JCM, Fong MW, et al. Impact assessment of non-pharmaceutical interventions against coronavirus disease 2019 and influenza in Hong Kong: an observational study. Lancet Public Health. 2020;5:e279–88. PubMed https://doi.org/10.1016/S2468-2667(20)30090-6

2. Cowling BJ, Ng DMW, Ip DKM, Liao Q, Lam WW, Wu JT, et al. Community psychological and behavioral responses through the first wave of the 2009 influenza A(H1N1) pandemic in Hong Kong. J Infect Dis. 2010;202:867–76. PubMed https://doi.org/10.1086/655811

3. Leung GM, Quah S, Ho LM, Ho SY, Hedley AJ, Lee HP, et al. A tale of two cities: community psychobehavioral surveillance and related impact on outbreak control in Hong Kong and Singapore during the severe acute respiratory syndrome epidemic. Infect Control Hosp Epidemiol. 2004;25:1033–41. PubMed https://doi.org/10.1086/502340

4. Leung GM, Ho LM, Chan SKK, Ho SY, Bacon-Shone J, Choy RY, et al. Longitudinal assessment of community psychobehavioral responses during and after the 2003 outbreak of severe acute respiratory syndrome in Hong Kong. Clin Infect Dis. 2005;40:1713–20. PubMed https://doi.org/10.1086/429923

5. Muth C, Oravecz Z, Gabry J. User-friendly Bayesian regression modeling: a tutorial with rstanarm and shinystan. Quant Methods Psychol. 2018;14:99–119. https://doi.org/10.20982/tqmp.14.2.p099

6. Borchering RK, Huang AT, Mier-Y-Teran-Romero L, Rojas DP, Rodriguez-Barraquer I, Katzelnick LC, et al. Impacts of Zika emergence in Latin America on endemic dengue transmission. Nat Commun. 2019;10:5730. PubMed https://doi.org/10.1038/s41467-019-13628-x

7. Gelman A, Hill J. Data analysis using regression and multilevel/hierarchical models. Cambridge: Cambridge University Press; 2007.

8. Vehtari A, Gelman A, Gabry J. Practical Bayesian model evaluation using leave-one-out cross-validation and WAIC. Stat Comput. 2017;27:1413–32. https://doi.org/10.1007/s11222-016-9696-4

9. Lee J, Gangnon RE, Zhu J, Liang J. Uncertainty of a detected spatial cluster in 1D: quantification and visualization. Stat (Int Stat Inst). 2017;6:345–59. https://doi.org/10.1002/sta4.161

10. Vehtari A, Simpson D, Gelman A, Yao Y, Gabry J. Pareto smoothed importance sampling. J Mach Learn Res. 2024;25:1–58

Appendix Table 1. The parameter estimates of the risk ratios change (exp(β)-1) and confidence intervals (CIs) from the three selected optimal models (not adjusted for weather)*

Formula	Avoid Crowded Places	Avoid Going Out as Much as Possible	Avoid Social Gathering	Wear Face Masks When Going Out	Wash Hands Immediately after Going Outside
lag_2 RSV_weekly_case ~ 1 + mask + avoid_crowd + avoid_goingout + wash_hand_after_outside + ct1 + offset(log(population))	−0.59 (−0.71, −0.42)	−0.62 (−0.72, −0.49)	NA	−0.35 (−0.50, −0.16)	−0.40 (−0.58, −0.13)
lag_2 RSV_weekly_case ~ 1 + mask + avoid_crowd + avoid_gathering + wash_hand_after_outside + ct1 + offset(log(population))	−0.58 (−0.70, −0.41)	NA	−0.62 (−0.72, −0.48)	−0.32 (−0.48, −0.11)	−0.39 (−0.57, −0.12)
lag_2 RSV_weekly_case ~ 1 + mask + avoid_goingout + avoid_gathering + wash_hand_after_outside + ct1 + offset(log(population))	NA	−0.58 (−0.69, −0.42)	−0.57 (−0.69, −0.41)	−0.32 (−0.48, −0.11)	−0.43 (−0.60, −0.18)

*In formulas, 1 is the intercept term. NA,.not available.

Appendix Table 2. The parameter estimates of the risk ratios change (exp(β)-1) and confidence intervals (CIs) from the selected optimal models (adjusted for weather)*

Formula	Avoid Crowded Places	Avoid Going Out as Much as Possible	Avoid Going to Health Care Facilities	Avoid Social Gathering	Wear Face Masks When Going Out	Wash Hands Immediately after Going Outside
lag_2 RSV_weekly_case ~ 1 + mask + avoid_crowd + avoid_goingout + wash_hand_after_outside + log_rel_hum + ct1 + offset(log(population))	−0.59 (−0.71, −0.43)	−0.63 (−0.73, −0.51)	NA	NA	−0.32 (−0.47, −0.11)	−0.40 (−0.59, −0.14)
lag_2 RSV_weekly_case ~ 1 + mask + avoid_crowd + avoid_goingout + wash_hand_after_outside + log_temp + log_rel_hum + ct1 + offset(log(population))	−0.60 (−0.71, −0.43)	−0.64 (−0.73, −0.50)	NA	NA	−0.32 (−0.47, −0.11)	−0.39 (−0.58, −0.13)
lag_2 RSV_weekly_case ~ 1 + mask + avoid_crowd + avoid_goingout + wash_hand_after_outside + power2_rel_hum + ct1 + offset(log(population))	−0.59 (−0.71, −0.43)	−0.63 (−0.73, −0.50)	NA	NA	−0.33 (−0.48, −0.13)	−0.40 (−0.58, −0.13)
lag_2 RSV_weekly_case ~ 1 + mask + avoid_crowd + avoid_goingout + wash_hand_after_outside + log_temp + log_abs_hum + ct1 + offset(log(population))	−0.59 (−0.71, −0.43)	−0.64 (−0.73, −0.51)	NA	NA	−0.31 (−0.47, −0.12)	−0.40 (−0.58, −0.15)
lag_2 RSV_weekly_case ~ 1 + mask + avoid_crowd + avoid_goingout + wash_hand_after_outside + power2_temp + power2_rel_hum + ct1 + offset(log(population))	−0.59 (−0.71, −0.42)	−0.63 (−0.73, −0.50)	NA	NA	−0.33 (−0.48, −0.13)	−0.40 (−0.58, −0.13)
lag_2 RSV_weekly_case ~ 1 + mask + avoid_crowd + avoid_gathering +	−0.59 (−0.71, −0.42)	NA	NA	−0.63 (−0.72, −0.50)	−0.28 (−0.45, −0.05)	−0.38 (−0.56, −0.11)

Formula	Avoid Crowded Places	Avoid Going Out as Much as Possible	Avoid Going to Health Care Facilities	Avoid Social Gathering	Wear Face Masks When Going Out	Wash Hands Immediately after Going Outside
wash_hand_after_outside + log_rel_hum + ct1 + offset(log(population))						
lag_2 RSV_weekly_case ~ 1 + mask + avoid_goingout + avoid_gathering + wash_hand_after_outside + log_rel_hum + ct1 + offset(log(population))	NA	−0.59 (−0.70, −0.44)	NA	−0.58 (−0.69, −0.42)	−0.28 (−0.45, −0.05)	−0.43 (−0.60, −0.19)
lag_2 RSV_weekly_case ~ 1 + mask + avoid_crowd + avoid_gathering + wash_hand_after_outside + log_temp + log_rel_hum + ct1 + offset(log(population))	−0.59 (−0.71, −0.43)	NA	NA	−0.63 (−0.72, −0.50)	−0.28 (−0.45, −0.05)	−0.38 (−0.56, −0.12)
lag_2 RSV_weekly_case ~ 1 + mask + avoid_goingout + avoid_gathering + wash_hand_after_outside + log_temp + log_rel_hum + ct1 + offset(log(population))	NA	−0.59 (−0.70, −0.44)	NA	−0.58 (−0.69, −0.42)	−0.28 (−0.44, −0.05)	−0.42 (−0.60, −0.18)
lag_2 RSV_weekly_case ~ 1 + mask + avoid_crowd + avoid_gathering + wash_hand_after_outside + power2_rel_hum + ct1 + offset(log(population))	−0.59 (−0.71, −0.42)	NA	NA	−0.62 (−0.72, −0.49)	−0.28 (−0.46, −0.06)	−0.38 (−0.57, −0.13)
lag_2 RSV_weekly_case ~ 1 + mask + avoid_goingout + avoid_gathering + wash_hand_after_outside + power2_rel_hum + ct1 + offset(log(population))	NA	−0.58 (−0.69, −0.44)	NA	−0.58 (−0.69, −0.42)	−0.28 (−0.45, −0.07)	−0.42 (−0.59, −0.19)
lag_2 RSV_weekly_case ~ 1 + mask + avoid_crowd + avoid_gathering + wash_hand_after_outside + power2_temp + power2_rel_hum + ct1 + offset(log(population))	−0.59 (−0.71, −0.41)	NA	NA	−0.63 (−0.72, −0.50)	−0.29 (−0.44, −0.08)	−0.38 (−0.56, −0.11)
lag_2 RSV_weekly_case ~ 1 + mask + avoid_crowd + avoid_goingout + wash_hand_after_outside + power2_temp + power2_abs_hum + ct1 + offset(log(population))	−0.59 (−0.71, −0.43)	−0.63 (−0.72, −0.51)	NA	NA	−0.34 (−0.49, −0.15)	−0.41 (−0.58, −0.15)
lag_2 RSV_weekly_case ~ 1 + mask + avoid_goingout + avoid_gathering + wash_hand_after_outside + power2_temp + power2_rel_hum + ct1 + offset(log(population))	NA	−0.58 (−0.70, −0.42)	NA	−0.58 (−0.69, −0.42)	−0.29 (−0.45, −0.08)	−0.42 (−0.60, −0.16)
lag_2 RSV_weekly_case ~ 1 + mask + avoid_crowd + avoid_goingout + wash_hand_after_outside + log_abs_hum + ct1 + offset(log(population))	−0.59 (−0.70, −0.42)	−0.63 (−0.72, −0.50)	NA	NA	−0.35 (−0.49, −0.16)	−0.41 (−0.59, −0.17)
lag_2 RSV_weekly_case ~ 1 + mask + avoid_crowd + avoid_goingout +	−0.59 (−0.71, −0.42)	−0.62 (−0.72, −0.49)	NA	NA	−0.35 (−0.50, −0.16)	−0.40 (−0.58, −0.13)

Formula	Avoid Crowded Places	Avoid Going Out as Much as Possible	Avoid Going to Health Care Facilities	Avoid Social Gathering	Wear Face Masks When Going Out	Wash Hands Immediately after Going Outside
wash_hand_after_outside + ct1 + offset(log(population))						
lag_2 RSV_weekly_case ~ 1 + mask + avoid_crowd + avoid_goingout + wash_hand_after_outside + power2_abs_hum + ct1 + offset(log(population))	−0.59 (−0.70, −0.42)	−0.62 (−0.72, −0.49)	NA	NA	−0.35 (−0.50, −0.17)	−0.41 (−0.59, −0.15)
lag_2 RSV_weekly_case ~ 1 + mask + avoid_crowd + avoid_goingout + wash_hand_after_outside + power2_temp + ct1 + offset(log(population))	−0.59 (−0.71, −0.42)	−0.62 (−0.72, −0.49)	NA	NA	−0.35 (−0.50, −0.16)	−0.41 (−0.58, −0.14)
lag_2 RSV_weekly_case ~ 1 + mask + avoid_crowd + avoid_goingout + wash_hand_after_outside + log_temp + ct1 + offset(log(population))	−0.58 (−0.70, −0.42)	−0.62 (−0.72, −0.49)	NA	NA	−0.36 (−0.50, −0.17)	−0.41 (−0.59, −0.17)
lag_1 RSV_weekly_case ~ 1 + mask + avoid_crowd + avoid_goingout + wash_hand_after_outside + log_rel_hum + ct1 + offset(log(population))	−0.57 (−0.69, −0.40)	−0.61 (−0.71, −0.47)	NA	NA	−0.36 (−0.50, −0.17)	−0.41 (−0.59, −0.16)
lag_1 RSV_weekly_case ~ 1 + mask + avoid_crowd + avoid_goingout + wash_hand_after_outside + log_temp + log_rel_hum + ct1 + offset(log(population))	−0.57 (−0.69, −0.40)	−0.61 (−0.71, −0.47)	NA	NA	−0.36 (−0.50, −0.18)	−0.41 (−0.59, −0.16)
lag_1 RSV_weekly_case ~ 1 + mask + avoid_goingout + avoid_gathering + wash_hand_after_outside + log_rel_hum + ct1 + offset(log(population))	NA	−0.56 (−0.68, −0.40)	NA	−0.57 (−0.68, −0.40)	−0.32 (−0.48, −0.12)	−0.43 (−0.60, −0.19)
lag_1 RSV_weekly_case ~ 1 + mask + avoid_goingout + avoid_gathering + wash_hand_after_outside + log_temp + log_rel_hum + ct1 + offset(log(population))	NA	−0.56 (−0.68, −0.40)	NA	−0.57 (−0.68, −0.41)	−0.32 (−0.48, −0.11)	−0.43 (−0.61, −0.18)

*In formulas, 1 is the intercept term, ‘log_’ means log transform, ‘power2_’ means quadratic transform. NA, not available.

Appendix Table 3. The parameter estimates of the risk ratios change of the model with a dummy variable indicating whether the year is in the duration of 2020 to 2022*

Formula	The dummy variable indicates the years 2020–2022	The RSV case of the previous week
RSV_weekly_case~1+d20_22+ct1+ offset(log(population))	−0.62 (−1.67, −0.57)	0.00 (0.00, 0.01)
RSV_weekly_case~1+d20_22+offset(log(population))	−0.76 (−0.81, −0.70)	NA

*In formulas, 1 is the intercept term, ct1 is the term of the one-week lag RSV case. NA, not available.

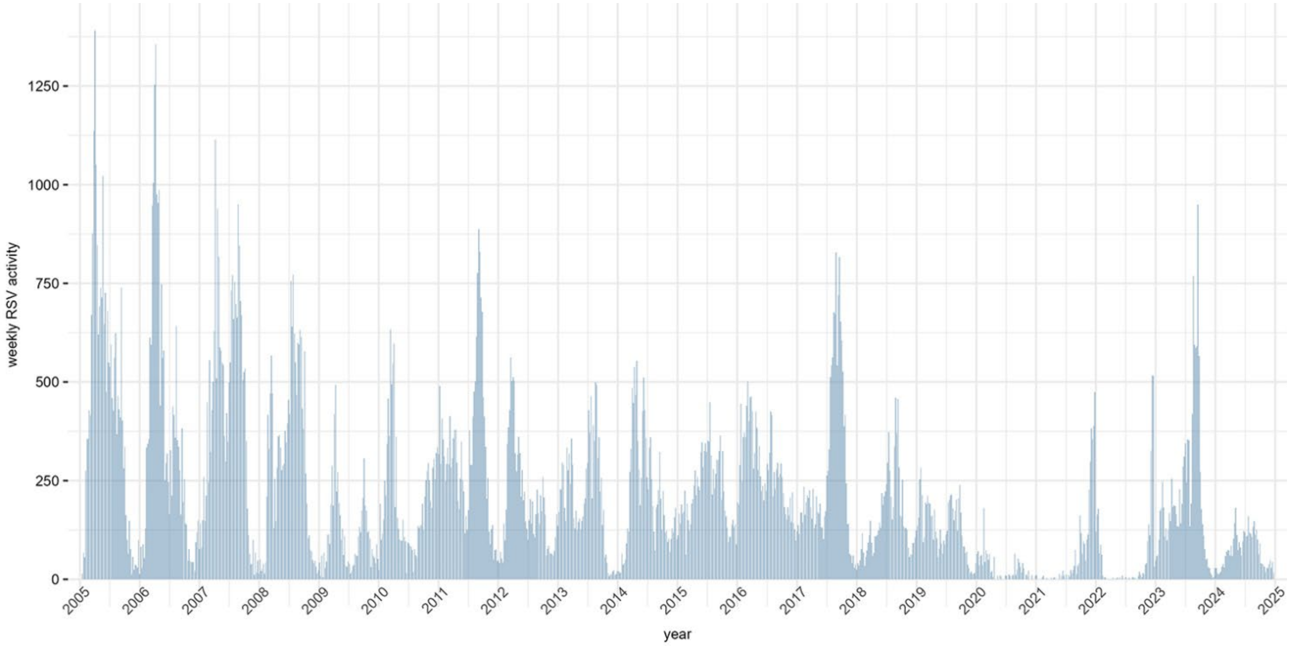

Appendix Figure 1. Weekly RSV activity from 2005 to 2024.

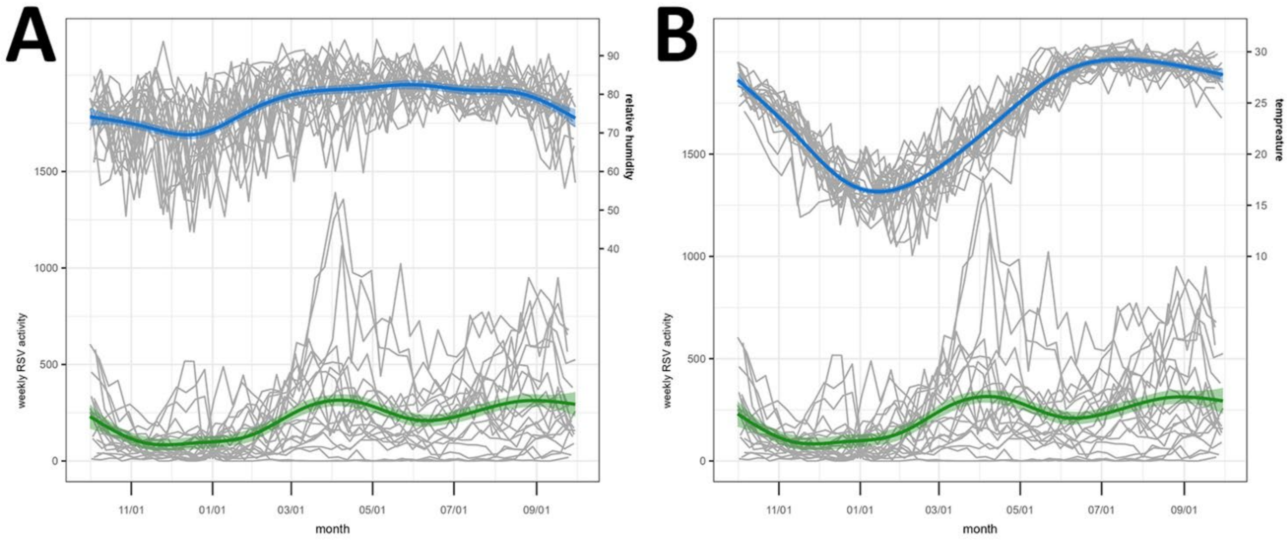

Appendix Figure 2. Estimated RSV activity and weather factors, (A) relative humidity and (B) temperature. The gray lines represent the coefficient multiplied by the log-transformed relative humidity and temperature, and the RSV activity in each year. The blue and green lines represent the smoothed conditional mean of the coefficient multiplied by the log-transformed relative humidity and temperature, and the RSV activity, respectively.

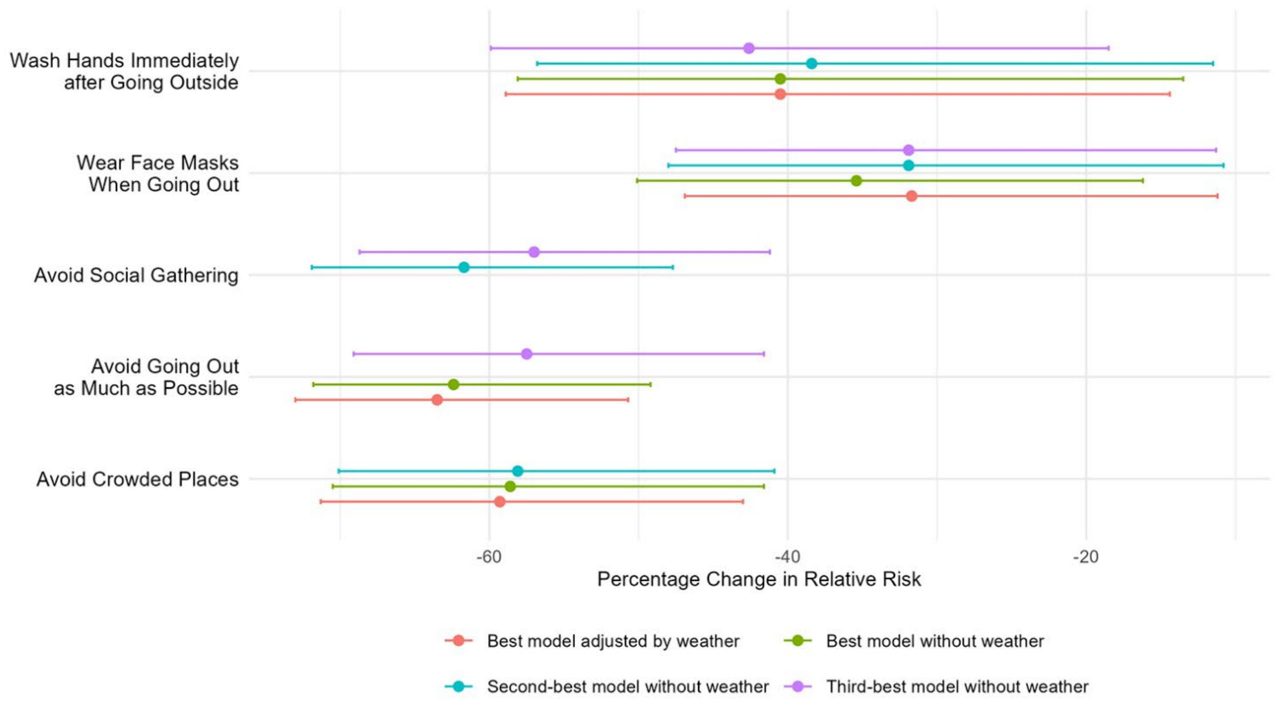

Appendix Figure 3. Percentage change in relative risk of the selected best models with and without the weather term.

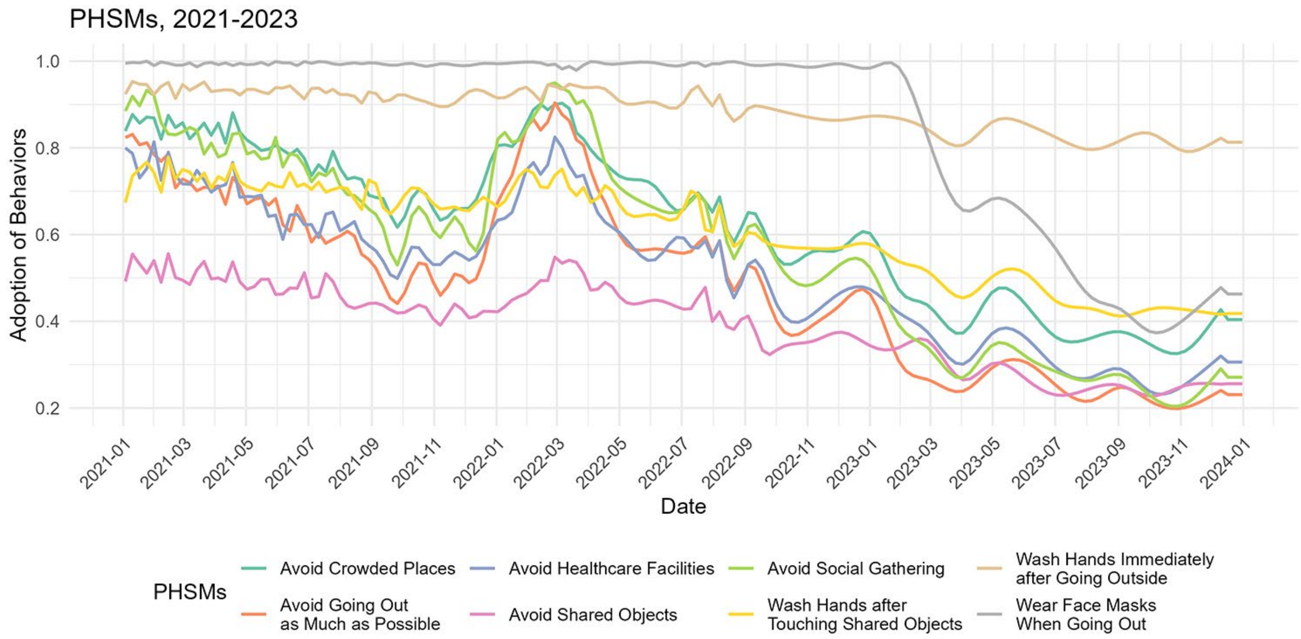

Appendix Figure 4. The trend of the PHSMs from 2021 to 2023.

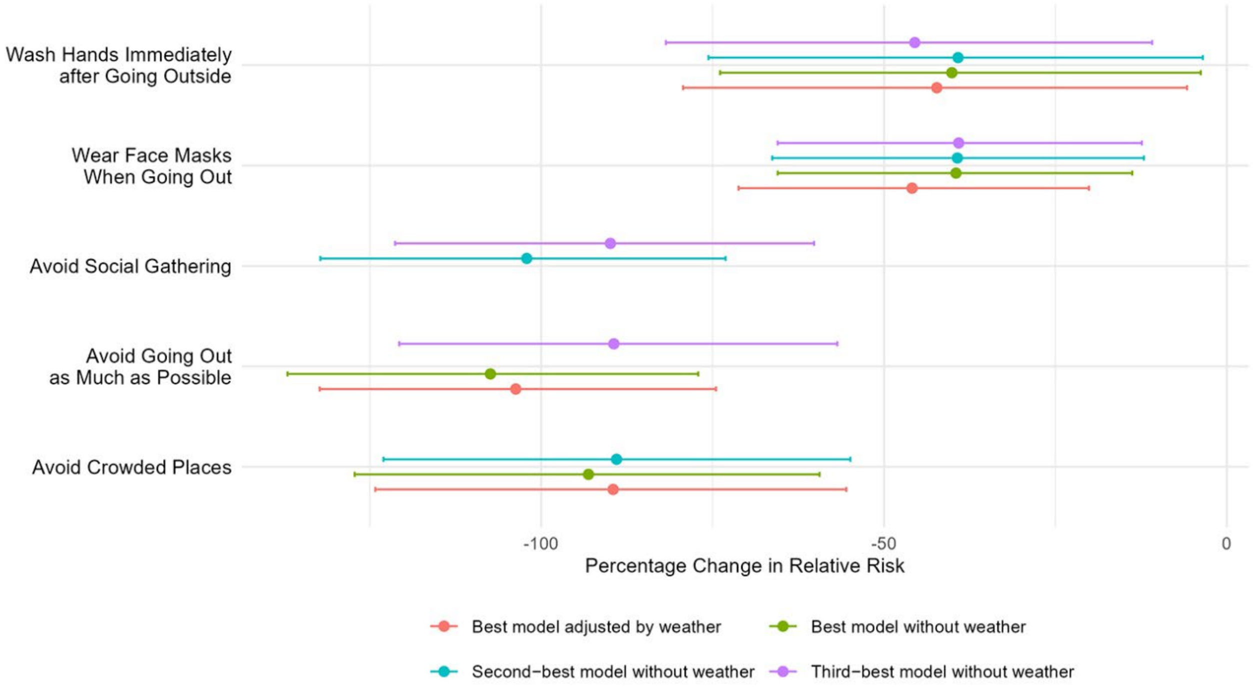

Appendix Figure 5. Percentage change in relative risk of the selected best models with and without the weather term adjusted by school closure.
